# Effect of silver diamine fluoride/potassium iodide treatment on the prevention of dental erosion in primary teeth: an in vitro study

**DOI:** 10.1038/s41405-023-00153-9

**Published:** 2023-07-06

**Authors:** Lamia Khairy Gadallah, Engie Mohamed Safwat, Reham Sayed Saleh, Shereen Musa Azab, Maha Moussa Azab

**Affiliations:** 1 Orthodontics and Pediatric Dentistry Department, National Research Centre, Giza, Egypt; 2 Restorative and Dental Materials Department, National Research Centre, Giza, Egypt; 3 Pharmaceutical Chemistry, Egyptian Drug Authority (EDA) (previous NODCAR), Giza, Egypt; 4 Department Pediatric Dentistry, Faculty of Dentistry, Fayoum University, Fayoum, Egypt; 5 Department of Pediatric Dentistry, School of Dentistry, New Giza University NGU, Giza, Egypt

**Keywords:** Oral diseases, Paediatric dentistry

## Abstract

**Introduction:**

Dental erosion has a great effect on oral health, when diagnosed it is irreversible, this sets the importance of different preventive measures being investigated against dental erosion.

**Aim:**

This in vitro study aims to evaluate the effectiveness of silver diamine fluoride and Potassium iodide **(**SDF-KI) in comparison to casein phosphopeptide-amorphous calcium phosphate fluoride (CPP-ACPF) varnish, sodium fluoride (NaF) varnish, silver diamine fluoride (SDF) alone and deionized water as a control group in the prevention of dental erosion in primary teeth and assessing its staining effect.

**Materials and methods:**

Forty deciduous teeth enamel specimens were randomly allocated into the five study groups. Tested materials were applied. An erosive challenge was done by immersing the specimens in a citric acid-containing soft drink with pH 2.85, for 5 min, 4 times/day, for 5 days. Changes in surface microhardness, mineral loss, and color change were evaluated besides recording of the surface topography and surface roughness for selected specimens.

**Results:**

The highest decrease in surface microhardness was recorded in the control group (−85.21 ± 10.60%), with a statistically significant difference (*p* = 0.002). SDF-KI group (−61.49 ± 21.08%) showed no statistically significant difference when compared to CPP-ACPF, NaF, and SDF groups. For calcium and phosphorous loss, control group was statistically significantly higher compared to the treatment groups (*p* = 0.003) and (*p* < 0.001) respectively, while there was no statistically significant difference between the tested treatment groups. The highest mean value for color change was recorded in SDF group (26.26 ± 10.31), followed by SDF-KI group (21.22 ± 12.87) with no statistically significant difference between groups.

**Conclusions:**

SDF-KI is as effective as CPP-ACPF, NaF varnishes and SDF in the prevention of dental erosion in primary teeth, there was no statistically significant difference regarding its staining potential.

## Introduction

Dental erosion is the irreversible loss of the tooth structure by the action of acids without the involvement of microorganisms [1, 2]. Dental erosion is more precisely related to the chemical process of softening and demineralization of the tooth structure while the term erosive tooth wear refers to a chemical-mechanical process where mechanical wear occurs to the softened structure [3]. The prevalence of erosion is about 30–50% for primary teeth and 20–45% for permanent teeth [4, 5].

The etiology of dental erosion could be related to external causative factors whether dietary due to excessive consumption of acidic foods and beverages, from drugs, or environmental sources [5, 6]. There are also some doubts concerning the relationship between a vegetarian diet and dental erosion that are not yet confirmed [3, 7, 8]. While the internal causative factors include gastroesophageal reflux disease (GERD), eating disorders such as bulimia, alcoholism, or associated with pregnancy [5, 6].

Primary teeth are more susceptible to the development and progression of dental erosion as the enamel is thinner and less mineralized, also salivary flow rates are lower for children, so it is essential to apply early and effective preventive measures [2].

Remineralizing agents such as fluorides are well proven to be effective in the prevention of dental caries as they can increase enamel hardness and resistance to acidic attacks [9]. However, in dental erosion, the acidic challenges are greater [6]. The efficacy of fluoride in the prevention of dental erosion needs to be furtherly investigated [10].

Silver diamine fluoride SDF has been proven effective in arresting dental caries due to the antibacterial property of the silver component, in addition to the role of fluoride in the prevention of mineral loss and remineralization of demineralized enamel and dentin [11, 12]. As for dental erosion, SDF is not yet widely studied [10, 13–15]. The application of potassium iodide KI following SDF was supposed to reduce the potential staining caused by SDF application by scavenging the free silver ions and forming a creamy white silver iodide precipitate [6].

Another well-known remineralizing agent is casein phosphopeptide-amorphous calcium phosphate (CPP-ACP) nano complex [16, 17]. It maintains a state of supersaturation of calcium and phosphate ions in plaque, so it prevents demineralization and enhances remineralization, it also interacts with fluoride ions to produce amorphous calcium phosphate fluoride (ACPF) phase [1]. The CPP-ACPF products have been shown recently to be more effective in the reduction of tooth surface loss [1, 16].

A single application of sodium fluoride varnish NaF has shown the potential to reduce enamel erosive challenges [1, 18–21]. They have high fluoride concentrations and act as fluoride reservoirs at the tooth surface [17].

Therefore, the aim of this in vitro study is to evaluate the effectiveness of SDF-KI in the prevention of dental erosion in primary teeth in comparison to CPP-ACPF, NaF varnishes, SDF and no treatment and investigate its staining effect. The null hypothesis was that SDF-KI has the same effect as SDF, NaF, and CPP-ACPF on the surface microhardness of enamel samples after being subjected to erosive cycling.

## Materials and methods

**Fig. 1:**
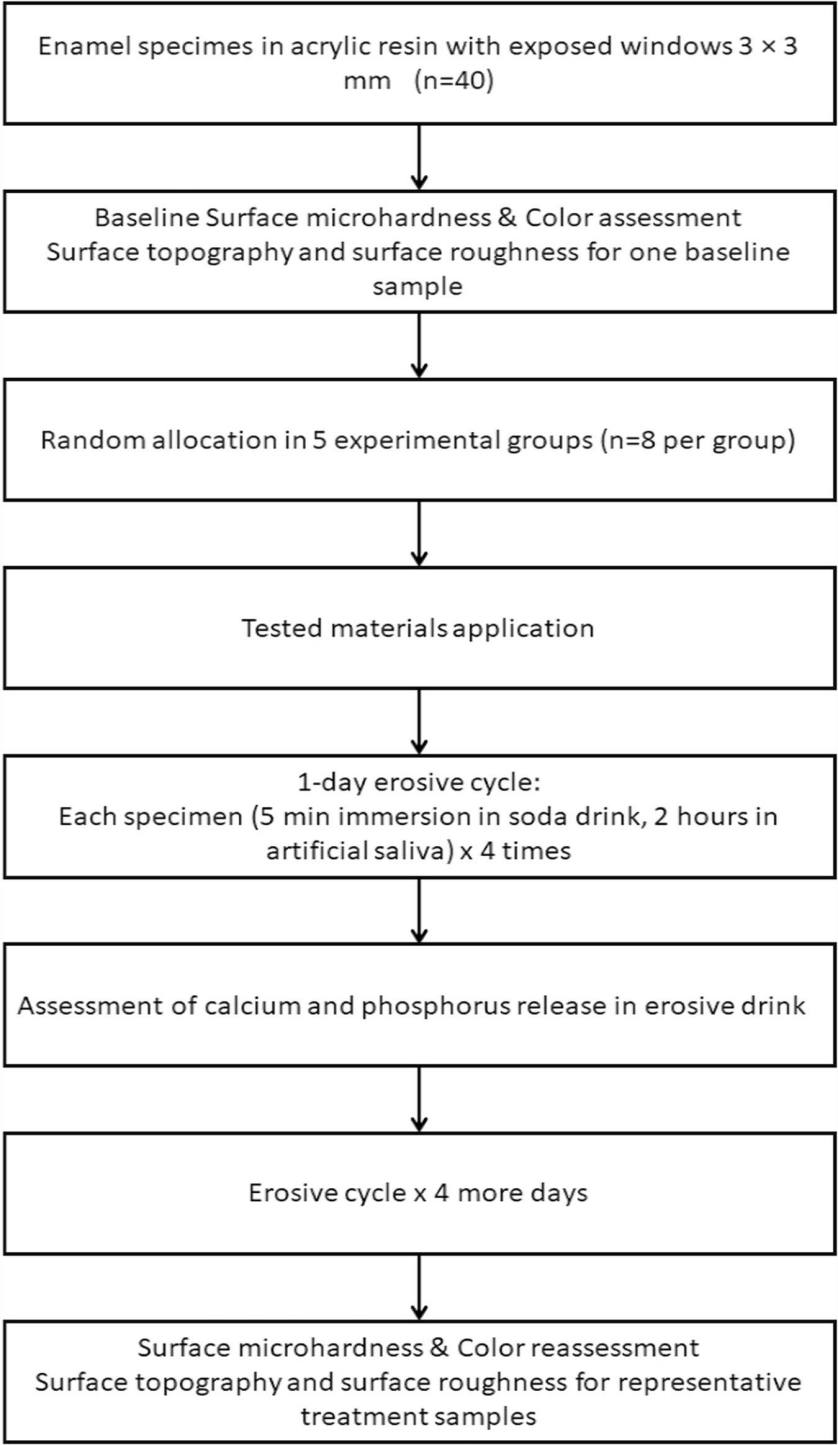
Study flow chart. Flowchart showing the laboratory steps of the study.

This study was approved by the Ethical Committee of the National Research Centre in Egypt under number 5433042021. The study flow chart is represented in Fig. 1.

### Sample size calculation

According to Gokkaya et al. [19], the mean value of surface microhardness was 263.5 ± 18.54 and 278.5 ± 21.4 for groups treated with NaF and CPP-ACPF, while according to Rallan et al. [16], the mean value of surface microhardness was 183.25 ± 3.21 and 209.36 ± 2.61 for fluoridated toothpaste and CPP-ACPF. A large effect size of approximately 0.73 is expected. Using G power statistical power analysis program (version 3.1.9.7) for sample size determination, A total sample size of 40 (8 in each group) will be sufficient to detect an effect size of approximately 1.04, with an actual power (1-β error) of 0.8 (80%) and a significance level (α error) 0.05 (5%) for the two-sided hypothesis test.

### Specimen preparation

After obtaining written informed consent from minor patients’ legal guardians, extracted primary molars were collected from the outpatient clinic, Faculty of Dentistry, Fayoum University. The age range for the children who provided the teeth is 5 years 6 months—11 years 8 months with a mean of 8 years 5 months. Collected molars were inspected carefully to be free of cracks, and developmental or acquired defects. Roots were separated from the crowns using diamond stone, remnants of dental pulp were excavated and discarded, then crowns were embedded in acrylic resin with the buccal or lingual enamel surfaces exposed. The exposed enamel surfaces of the specimens were ground flat using wet waterproof fine silicon-carbide abrasive paper with grit sizes 400, 600, and 1000, specimens were then rinsed in deionized water. An acid-resistant nail varnish (Colorama Maybelline™, Cosbra Cosmetics Ltda., Brazil) was applied on the enamel surface except for a 3 mm × 3 mm window left exposed. Finally, specimens were stored in deionized water.

### Randomization and allocation

Teeth were randomly allocated using *random.org* into five groups,1: SDF-KI, 2: CPP-ACPF, 3: NaF, 4: SDF, 5: Deionized water as a control group (Table 1).

**Table 1:** Study groups.

Group	Product	Active ingredient	Trade name	Manufacturer
Group 1	38% SDF + KI solution	38% silver diamine fluoride (44,800 ppm F^−^) + saturated potassium iodide solution	Riva Star Step 1 + Riva Star Step 2	SDI Limited, Australia
Group 2	CPP-ACPF varnish	5% sodium fluoride (22,600 ppm F^−^), 2% casein phosphopeptide-amorphous Calcium Phosphate	MI Varnish	GC Europe
Group 3	NaF varnish	5% sodium fluoride (22,600 ppm F^−^)	ProShield	PD, Germany
Group 4	38% SDF solution	38% silver diamine fluoride (44,800 ppm F^−^)	Riva Star Step 1	SDI Limited, Australia
Group 5	No treatment (Deionized water)	n/a	n/a	n/a

#### Sample treatment

The tested materials were applied following manufacturer instructions. NaF and CPP-ACPF groups: varnishes were applied with a micro brush to produce an even layer on a clean dry enamel surface. SDF group: SDF solution was applied to the enamel using a micro brush, and excess material was blotted away. SDF-KI group: SDF solution was applied to enamel using a micro brush, then KI solution was immediately applied using a micro brush and it was continuously applied until the initially creamy white surface turns clear, then excess material was blotted away, while for the control group, no treatment was applied.

Specimens were then stored in artificial saliva for 24 h, artificial saliva (2.38 g Na_2_HPO_4_, 0.19 g KH_2_PO_4_ and 8.00 g NaCl per liter of distilled water adjusted with phosphoric acid to pH 6.75, 10 mL/specimen) was prepared according to Karavana et al. study [22], then the tested varnishes in groups 2 and 3 were removed carefully using a scalpel blade and acetone before the erosive challenge [17].

#### Erosive challenge

Specimens were immersed in a soft drink (Seven-up, Egypt, pH 2.85) that contains citric acid for 5 min, 4 times/day, using separate containers (10 mL/specimen), the specimens were rinsed thoroughly with deionized water and immersed in artificial saliva for 2 h, between each immersion in soft drink and overnight. This erosive cycle was repeated for 5 days [17]. The soft drink and artificial saliva were refreshed at the beginning of each cycle. The whole erosive cycle took place at room temperature, where specimens were always kept in hermetically sealed containers to prevent the loss of gas, which can increase the pH.

#### Surface microhardness measurement

Baseline enamel and post-erosive challenge surface microhardness were measured for each specimen using a Digital Vickers hardness tester (NEXUS 4000^TM^, INNOVATEST model No 4503, Netherland). For the testing, three indentations were performed for each specimen with a load of 100 g, for 10 s dwell time, at magnification ×40 [23]. The mean of the three values was recorded and presented as Vickers hardness number (VHN).

The percent change in surface microhardness was calculated by the formula:

(VHN post − VHN baseline)/VHN baseline × 100 [17]

### Measurement of mineral loss

The amount of calcium and phosphorus released into the erosive solution from each sample was measured using a spectrophotometer (Shimadzu spectrophotometer, Japan) with the aid of a specific reagent for each element. At the end of the first one-day erosive cycle, the erosive solution of each sample + the specific detection reagent is placed in a cell in the spectrophotometer, the other cell will contain the unused erosive solution + the specific detection reagent. The spectrophotometer will detect the difference in the concentration of calcium and phosphorus between the 2 cells.

### Color change measurement

Baseline and post-erosive challenge color of enamel specimens were evaluated with the VITA Easyshade® advance portable dental spectrophotometer (VITA Zahnfabrik GmbH, Bad Säckingen, Germany) before the application of the tested materials and after the end of the erosive challenge.

Color assessment was done according to the Commission International del’Eclairage L*a* b* color system. The L* (lightness from black to white), a* (red to green), b* (yellow to blue), h (hue) and C (chroma) were evaluated 3 times, and the average measurements were recorded.

The change in the color (ΔE) in groups was calculated based on the mathematical equation of ΔE* = [(Δ*L*) ^2^ + (Δ*a*) ^2^ + (Δ*b*) ^2^] ^1/2^ [24].

### Surface topography and Surface roughness

For surface topography, two-dimensional and three-dimensional images were recorded using Atomic Force Microscope AFM (Anton Paar - Tosca™ 200, USA). Four images were taken for a baseline specimen before pH cycling as well as for representative specimens for each treatment group after pH cycling. A scanning size of 10 µm × 10 µm with a resolution 400 × 400 was acquired using Arrow NCR tapping cantilever. For surface roughness, different height parameters were measured and root mean square heights (Sq) were recorded, and the AFM data were processed according to ISO 25178 using Tosca analysis software specialized program [1, 17].

### Statistical analyses

Data management and statistical analysis were performed using the Statistical Package for Social Sciences (SPSS) version 18. Numerical data were summarized using means, standard deviations, minimum, maximum, and confidence intervals. Data were explored for normality using Kolmogorov-Smirnov. Data were compared between groups using one-way analysis of variance (ANOVA) test, followed by Tukey’s post hoc test if the difference between groups was found to be significant. Comparison within the same group was performed using paired t-test.

All *p* values are two-sided. *P* values ≤ 0.05 were considered significant.

## Results

Comparisons between groups concerning normally distributed numeric variables for surface microhardness, mineral loss and color change were compared by one-way analysis of variance (ANOVA).

**Fig. 2:**
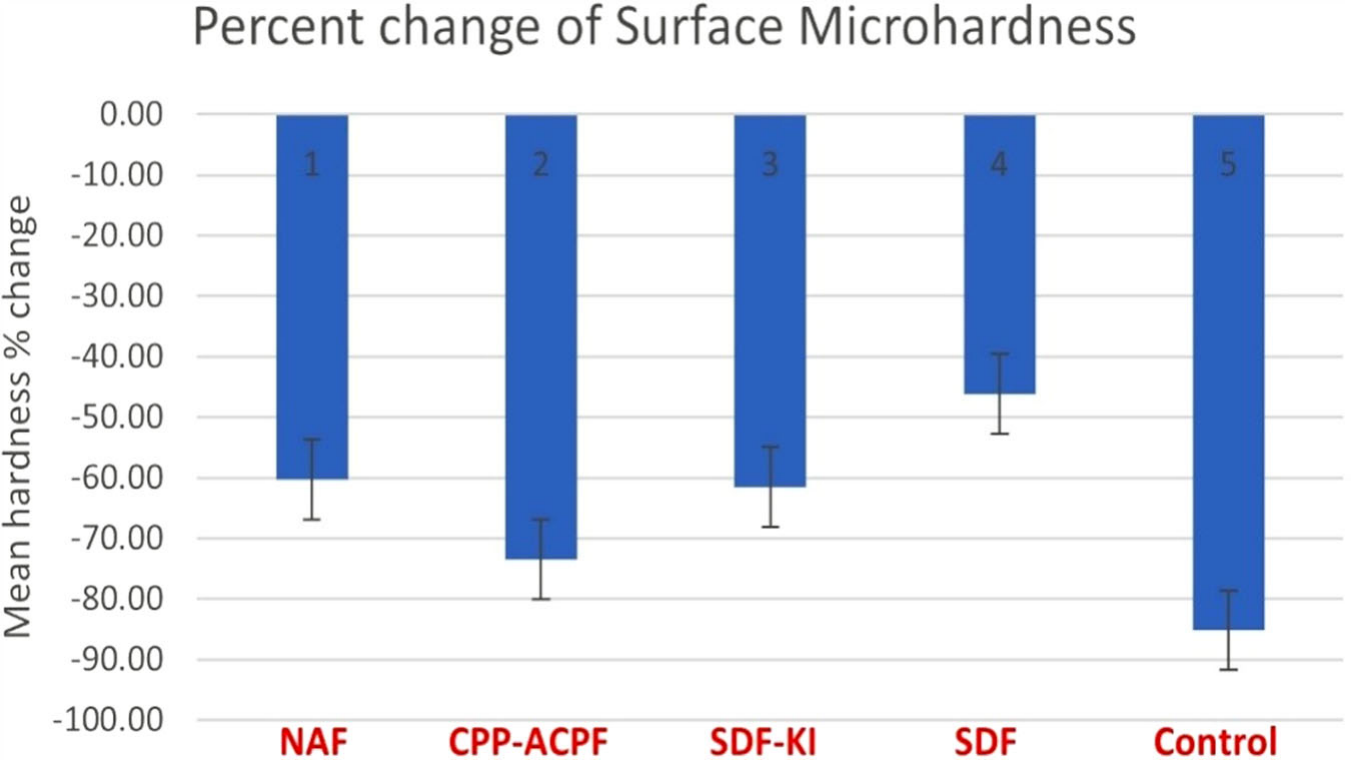
Surface microhardness. Bar chart for percent change in surface microhardness for study groups.

For surface microhardness, there was no significant difference between groups (*p* = 0.675) for baseline mean values. The highest percent decrease was recorded in the control group (−85.21 ± 10.60%), followed by the CPP-ACPF group (−73.52 ± 9.37%), then the SDF-KI group (−61.49 ± 21.08%), then the NaF group (−60.32 ± 28.35), with the lowest decrease recorded in the SDF group (−46.17 ± 11.89). ANOVA test and Tukey’s post hoc test showed that the percent change of the control group was statistically significantly higher than other experimental groups (*P* = 0.002), but there was no statistically significant difference between SDF-KI, NaF, CPP-ACPF, and SDF groups (Table 2). For comparisons within the same group using paired t-test, the mean values within all groups showed a statistically significant decrease (*p* = 0.00) after erosion as shown in (Table 2 and Fig. 2).

**Table 2:** Descriptive statistics and percent change in surface microhardness between study groups.

% of change	Mean	SD	SE	95% Confidence interval for mean		Min	Max	*F*	*p* value
				Lower bound	Upper bound				
NaF	−60.32^a,b^	28.35	10.02	−84.02	−36.62	−87.8	−1.14	5.468	0.002
CPP-ACPF	−73.52^b,c^	9.37	3.31	−81.36	−65.69	−82.57	−53.36		
SDF-KI	−61.49^a,b,c^	21.08	7.45	−79.12	−43.87	−82.51	−27.02		
SDF	−46.17^a^	11.89	4.20	−56.11	−36.22	−64.33	−32.08		
Control	−85.21^c^	10.6	3.75	−92.56	−77.87	−97.07	−62.85		

Different letters (a, b, c) within the mean column indicate significant difference at *p* < 0.05 (Tukey’s HSD).

As for mineral loss, the mean value of Ca ion release [conc.in M (×10^−3^)] was the highest for the control group (1.33 ± 0.06). ANOVA test and Tukey’s post hoc test showed that the mean value recorded in the control group was statistically significantly higher compared to other experimental groups (*p* = 0.003). The mean values recorded for calcium ion release were the lowest in the SDF-KI group (0.69 ± 0.34) followed by the NaF group (0.71 ± 0.43), then CPP-ACPF group (0.88 ± 0.46), and the SDF (0.97 ± 0.08). However, the difference between the NaF, CPP-ACPF, SDF and SDF-KI groups didn’t reach the level of statistical significance (Table 3).

**Table 3:** Descriptive statistics and comparison between groups regarding Calcium and Phosphorous concentration [M (×10^−3^)] (ANOVA test).

		Mean	SD	SE	95% Confidence interval for mean		Min	Max	*F*	*p* value
					Lower bound	Upper bound				
Calcium	NaF	0.71^b^	0.43	0.15	0.35	1.07	0.13	1.29	5.064	0.003
	CPP-ACPF	0.88^b^	0.46	0.16	0.50	1.27	0.23	1.55		
	SDF-KI	0.69^b^	0.34	0.12	0.40	0.97	0.29	1.41		
	SDF	0.97^a,b^	0.08	0.03	0.90	1.04	0.88	1.09		
	Control	1.33^a^	0.06	0.02	1.28	1.38	1.26	1.42		
Phosphorus	NaF	1.27^b^	0.13	0.05	1.16	1.38	1.12	1.50	48.89	<0.001
	CPP-ACPF	1.27^b^	0.23	0.08	1.08	1.47	1.02	1.62		
	SDF-KI	1.21^b^	0.08	0.03	1.14	1.27	1.07	1.34		
	SDF	1.02^b^	0.29	0.10	0.79	1.27	0.63	1.50		
	Control	2.61^a^	0.42	0.15	2.26	2.96	1.86	3.01		

Within the same comparison, different letters (a, b) within the mean column indicate significant difference at *p* < 0.05 (Tukey’s HSD).

For phosphorus ion release [conc. in M (×10^−3^)], the highest mean value was recorded in the control group (2.61 ± 0.420), followed by the CPP-ACPF group (1.27 ± 0.23), then NaF group (1.27 ± 0.13), then SDF-KI group (1.21 ± 0.08) with the lowest mean value recorded in the SDF group (1.02 ± 0.29). ANOVA test and Tukey’s post hoc test revealed that the mean value recorded in the control group was significantly higher compared to the experimental groups (*p* < 0.001). However, the difference between the NaF, CPP-ACPF and SDF-KI groups didn’t reach the level of statistical significance (Table 3).

**Fig. 3:**
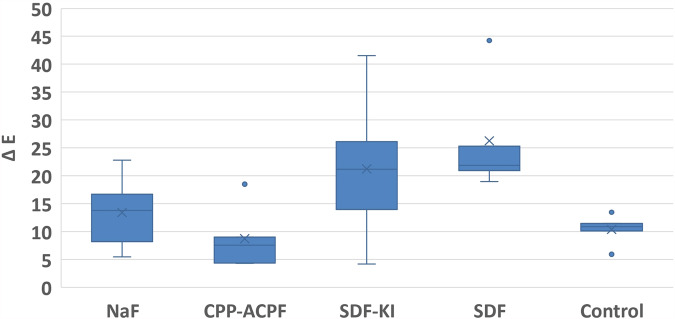
Color change. Box plot for color change in study groups.

As for color change measurements, the highest mean value was recorded in SDF group (26.26 ± 10.31), followed by SDF-KI group (21.22 ± 12.87), then NaF group (13.39 ± 6.88), then the control group (10.37 ± 2.77), with the lowest mean value recorded in CPP-ACPF group (8.37 ± 5.84). The difference between groups was not statistically significant (*p* = 0.02), (shown in Fig. 3).

**Fig. 4:**
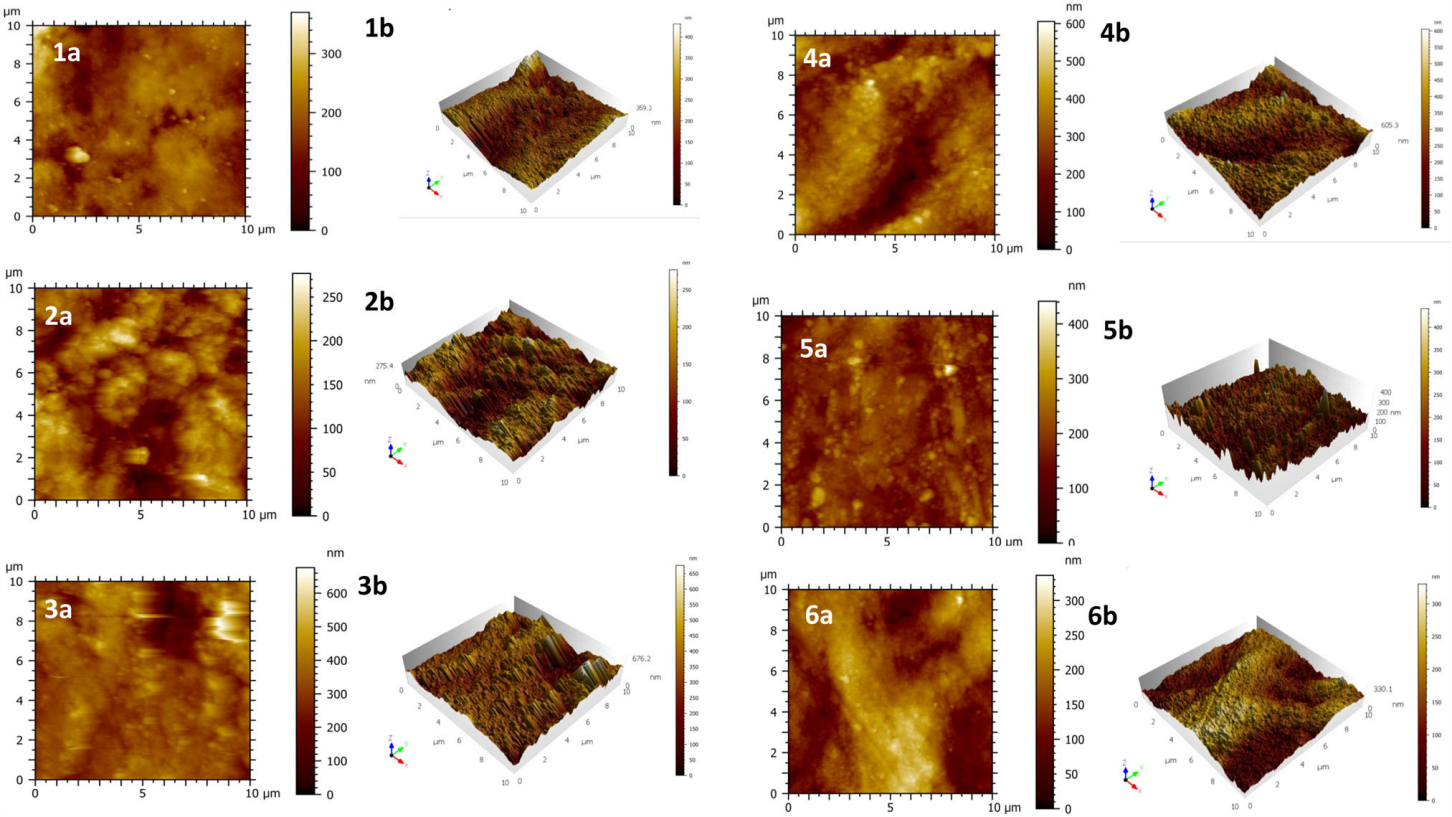
Surface topography. AFM two-dimensional and three-dimensional images for a representative sample of the baseline and study groups.

AFM two-dimensional and three-dimensional images clearly demonstrated the effect of the treatment products on the topography of the enamel surface. The baseline specimens (Fig. 4:1a,1b) before any pH cycling exhibited homogeneous and uniform surfaces, however, the control specimen after the acidic challenge (Fig. 4:2a) showed a honeycomb-like structure, with exposure of enamel prism and enamel interprismatic structures. The CPP-ACPF image (Fig. 4:3a) showed a more homogenous appearance compared to NaF image (Fig. 4:4a,4b) which showed greater depressions, while the SDF-KI image (Fig. 4:6a,6b) showed lower depressions and a smoother surface.

For surface roughness the mean value of the four readings of Sq for each group was the highest for the control group (80.75 ± 15.86), followed by the NaF group (76.89 ± 24.69), then the CPP-ACPF group (62.84 ± 30.6), then the SDF-KI group (49.89 ± 5.48) and the lowest mean value was for the SDF group (46.09 ± 9.48), while the baseline mean value was (48.11 ± 13.15).

## Discussion

High prevalence rates of dental erosion had drawn clinicians’ and researchers’ attention to this problem [10, 13, 25]. The increased consumption of acidic soft drinks and energy drinks especially among adolescents and children established the importance of effective preventive measures [26].

Different fluoride presentations were tested against dental erosion as toothpaste, rinses, gels, and varnishes [3, 18, 20, 27].

The rise of SDF as an effective anti-cariogenic agent, their role in the prevention of mineral loss, and increasing the resistance of dental hard tissues to acids lead us to study their effect on dental erosion [11, 12, 28]. The effectiveness of SDF- KI is less than SDF alone in the prevention of caries progression as KI reduces the number of silver ions that have antimicrobial properties, but this effect is irrelevant to dental erosion due to the absence of bacterial component [29].

To our knowledge, this is the first study to evaluate the effect of SDF-KI in the prevention of dental erosion in primary teeth; we studied its effect versus CPP-ACPF, NaF and SDF alone. We kept the comparisons between fluoride-containing products for the treatment groups as the effect of dental erosion without fluoride is overwhelming [1, 18, 19, 21]. The SDF group was added to compare its effect with and without the addition of KI.

The choice of CPP-ACPF was due to its ability to protect against acidic attacks and its remineralization potential [16]; however, its effect against erosion is still controversial [17, 26]. CPP-ACPF is different from any other fluoride-containing preventive measure in the high availability of calcium and phosphate, so the preventive action of fluoride is aided by the high uptake of calcium and phosphate from the surrounding environment in the presence of CPP-ACPF [30].

As for NaF, it is the most commonly used fluoride salt and it has shown an effective preventive role so we used the 5% NaF varnish for comparison [18, 21, 31].

For those two comparator groups of CPP-ACPF and NaF, varnishes were chosen over other forms of fluoride as it is commonly used with the young age, it offers high fluoride concentrations and a relatively long adherence and fluoride release time to the tooth surface and both factors are required to offer better protection against lower concentrations of erosive acids [3, 21].

Trying to mimic the clinical situation the varnish was applied only once and removed after 24 h as this might occur in the oral cavity by tooth brushing and other mechanical actions, so their chemical rather than their physical protective effect was tested [17, 18].

For the evaluation of dental erosion, several techniques had been used both in vivo and in vitro [18]. As an accurate and reliable technique, surface microhardness has been used frequently to assess dental erosion in vitro [17, 19, 23].

The differences in surface microhardness values between the SDF-KI, NaF and CPP-ACPF groups showed no statistically significant difference. The lowest reduction in surface microhardness was in the SDF group. The control group showed a statistically significant difference in microhardness compared to the treatment groups of SDF and NaF. Similarly, Suresh et al. reported a statistically significant difference in microhardness in favor of SDF compared to the control group [14], also Gokkaya et al. study [12] showed a statistically significant decrease in microhardness of the control group in comparison to the treatment groups of NaF and CPP-ACPF varnishes and showed a nonsignificant difference in surface microhardness reduction between the CPP-ACPF varnish and NaF varnish. While a statistically significant difference in microhardness in favor of CPP-ACPF paste in comparison to fluoride varnish was previously reported in Carvalho et al. study [17].

The lowest decrease in surface microhardness in the SDF group could be attributed to the high fluoride concentration with the synergic effect of high silver ions that can increase surface hardness by forming silver phosphate and insoluble metallic silver salts [12–14]. However, there was no statistically significant difference with the SDF-KI on the percent change of surface microhardness despite of the scavenging of silver ions. The privilege of high fluoride concentrations is still present in the professionally applied varnishes as NaF and CPP-ACPF and that explains why there was not a statistically significant difference between the treatment groups. Their effect against the erosive challenge is due to the establishment of the protective layer CaF–like layer on the enamel surface and the formation of fluorapatite crystals [14, 26]. On the other hand, the additional remineralizing effect of calcium and phosphate in the CPP-ACPF was not evident in these results.

The evaluation of dental erosion by calcium and phosphorus loss using spectrophotometry has been considered another efficient and sensitive method [10]. Unsurprisingly, the control group showed the highest results in calcium and phosphorous loss in comparison to the treatment groups with a statistically significant difference.

The results for phosphorous loss showed that the SDF group showed the lowest values although not statistically significant than the other treatment groups, this positive effect of SDF in the prevention of phosphorous loss was recorded in a previous study [10] where 10%SDF group showed a statistically significant difference from 2%NaF solution group with a high acidic challenge of 10%citric acid, while in the milder challenge of 1% citric acid, both agents were effective with no statistically significant difference. One explanation could be the formation of silver phosphate on the enamel surface due to the reaction with silver ions in SDF while the addition of KI in the SDF-KI group affected the silver ions.

As for calcium loss, all treatment groups showed no statistically significant difference between them. Similarly, the comparison between NaF and CPP- ACPF varnishes showed no statistically significant difference before, concerning calcium loss [19], although both groups showed statistically significantly lower values than the negative control group of deionized water. Both agents had a protective effect against dental erosion but with no superiority of one over the other [1, 19].

Measuring surface roughness is an established approach for erosion testing [1, 17], this method provides a very descriptive presentation of the erosion effect, in this study AFM was used to study the topography of the etched and the treated enamel surface, mean Sq of each group was presented as an estimation of the surface roughness and accordingly, the erosive effect of the tested groups.

The AFM results were compatible with the surface microhardness results. They showed that all the tested materials decreased the surface roughness compared to the control group after the erosive challenge.

The surface microhardness and mineral loss results as well as the AFM images showed that those tested fluoride products offered some degree of protection but not complete prevention against dental erosion in accordance with previous studies [10, 13, 19, 21, 32].

Considering that the major concern for the use of SDF is its staining potential so the color changes were assessed in this study. The assessment of color changes by the naked eye is often subjective, so the device-based measurements using the spectrophotometer were chosen as an objective and more precise method [29].

The color change assessment showed no statistical difference between all groups, whereas the highest mean value was recorded in the SDF group expectedly followed by the SDF-KI group. These results were similar to a previous study where SDF-KI caused perceptible staining when used for recurrent caries prevention adjacent to restorations [29].

In the current study, we tried to mimic the intra-oral environment as far as possible; however, some limitations were present, as in the oral cavity erosion never occurs solo, it is usually superimposed by abrasion due to food chewing or tooth brushing. Further studies with larger sample sizes and in vivo settings are required for a better evaluation of the clinical effectiveness of SDF-KI in the prevention of dental erosion and its associated risk of teeth discoloration.

## Conclusions

Within the limitation of this study, SDF-KI revealed comparable effectiveness in the prevention of dental erosion in primary teeth as CPP-ACPF, NaF varnishes and SDF, additionally, there was no statistically significant difference regarding its staining potential.

### Benefits of the findings of the paper

- This study shed light on one of the indications of SDF-KI in erosion prevention.
- Practitioners will have an idea about the degree of prevention they would get from a high-concentration fluoride preparation.
- The addition of KI to SDF does not completely solve the discoloration problem.

## Data Availability

The datasets used and/or analyzed during the current study are available from the corresponding author on reasonable request.
